# Assessment of the Level of Knowledge About Risk Factors, Prevention, and Treatment of Gestational Diabetes Mellitus in a Community Sample From Saudi Arabia

**DOI:** 10.7759/cureus.58435

**Published:** 2024-04-17

**Authors:** Suzan A Morsy, Ayat M Tawfik, Samar Y Badayyan, Lameer K Shaikh, Shaden AzizKhan, AlKhansaa A Zakari

**Affiliations:** 1 Department of Pathological Sciences, Fakeeh College for Medical Sciences, Jeddah, SAU; 2 Department of Clinical Pharmacology, Faculty of Medicine, Alexandria University, Alexandria, EGY; 3 Department of Clinical Sciences, Fakeeh College for Medical Sciences, Jeddah, SAU; 4 Department of Medicine and Surgery, Fakeeh College for Medical Sciences, Jeddah, SAU

**Keywords:** diagnosis, complications, risk factors, awareness, gestational diabetes mellitus (gdm), kingdom of saudi arabia (ksa)

## Abstract

Introduction

Gestational diabetes mellitus (GDM) is a common disease affecting pregnant females, and it carries a major risk of short and long-term health problems for both mothers and their offspring. Multiple factors like advanced maternal age, obesity, and unhealthy lifestyle can increase the risk of GDM. The current guidelines recommend screening all pregnant females for risk factors during the first trimester with subsequent testing of the blood glucose level at 24 weeks gestation. Lack of awareness about GDM is a main contributing factor in the delay in screening and diagnosis of GDM with subsequent fetal and maternal complications. This study aims to identify the level of knowledge about GDM among the adult population in the Kingdom of Saudi Arabia (KSA).

Material and methods

A descriptive cross-sectional questionnaire-based study was conducted to identify the level of knowledge about risk factors, prevention, and treatment of GDM in a community sample from Saudi Arabia. A self-administered electronic questionnaire was designed, tested for validity and reliability, and distributed through social media platforms. It consisted of 18 questions asking about the socio-demographic characteristics, the type of hospital in which the participant receives their medical care, whether the participant heard about GDM or not, and if they know someone with GDM, in addition to questions to assess the level of knowledge about risk factors, complications, prevention, and treatment of GDM. The total score of knowledge was calculated. The multivariate regression analysis test was employed to analyze the relationship between various demographic variables and the level of knowledge about GDM among the study population. A p-value of 0.05 or less was considered statistically significant.

Results

A total of 539 (100%) participants completed the questionnaire: 263 (48.8%) of them were in the age category (18-25 years), 440 (81.6%) of them were females, 307 (57%) had a bachelor's degree, 275 (51%) were single, 454 (84.2%) had heard about GDM, and 258 (47.9%) of them have or know someone with GDM. The total score of knowledge revealed excellent, good, fair, and poor levels among 334 (62%), 140 (26%), 49 (9%), and 16 (3%) of participants, respectively. The multivariable linear regression model revealed that participants who received health care from governmental hospitals heard about GDM and had or knew someone with GDM were positively associated with a higher level of knowledge.

Conclusions

The findings revealed that among participants, 62% showed excellent knowledge about GDM, although, the other 38% had non-optimal levels of knowledge. Awareness campaigns are recommended to improve the level of knowledge about this disease, its risk factors, treatment, and complications.

## Introduction

Gestational diabetes mellitus (GDM) is defined as any degree of glucose intolerance that is first recognized during pregnancy. Sometimes it can result in lethargy, polyuria, nausea, and increased risk of infections, like vaginal and urinary tract infections; however, it is more common to be asymptomatic [1]. The prevalence of GDM has obviously increased in the last decade [2]. In the Kingdom of Saudi Arabia (KSA), it is estimated to affect about 11.7% of pregnant females [3]; thus, it carries a major risk of short and long-term health problems for both mothers and their offspring [4].

Health-related risks that are increased among mothers with GDM include pregnancy-related complications, like abortion, preeclampsia, delivery of low-birth-weight infants, macrosomia, stillbirth, and vaginal lacerations, postpartum complications, such as postpartum hemorrhage, and depression, in addition to delayed complications like type 2 diabetes mellitus (T2DM), and cardiovascular diseases. On the other side, newborn complications also are increased, including short-term complications like neonatal hypoglycemia, hyperbilirubinemia, shoulder dystocia, and birth trauma, in addition to delayed complications like higher risk of developing obesity, T2DM, cardiovascular disease, and other metabolic diseases [5].

Multiple risk factors can increase the probability of developing GDM, including non-modifiable risk factors like ethnicity, genetic polymorphisms, and advanced maternal age, in addition to family history of GDM. On the other hand, modifiable risk factors such as a high body mass index before conception, weight gain during pregnancy, a westernized diet, insulin resistance, and polycystic ovarian syndrome. Each of these risk factors is linked, either directly or indirectly, to decreased insulin sensitivity and/or β-cell activity, which results in poor glucose tolerance and hyperglycemia [6].

The current guidelines recommend screening all pregnant females for risk factors during the first trimester; if no risk factors are identified, a two-hour 75 gr oral glucose tolerance test is recommended to be done at 24 weeks gestation [7].

The best approach to minimize GDM-associated complications is to prevent the development of this disease through the identification and modification of the risk factors even before conception. Also, appropriate awareness of the whole community about GDM helps to ensure appropriate screening of pregnant females, with subsequent early identification of females with/or at risk of developing GDM and early intervention and monitoring to minimize maternal and fetal complications. However, lack of awareness about GDM is a main contributing factor in the delay in screening and diagnosis of GDM with subsequent risk of feto-maternal morbidity and mortality [8].

The current study aims to identify the level of knowledge about risk factors, prevention, and treatment of GDM in a community sample from Saudi Arabia.

## Materials and methods

Study design and setting

A descriptive cross-sectional questionnaire-based study was conducted among the adult population in Saudi Arabia between December 2020 and September 2023.

Participants, inclusion, and exclusion criteria

Participants were recruited using volunteer sampling.

The inclusion criteria included being adults (aged 18 years or older), mentally competent individuals located in Saudi Arabia, literate (capable of reading and writing in Arabic or English), and willing to participate in this study.

Exclusion criteria were respondents with a language barrier hindering them from communicating with Arabic or English languages, healthcare professionals, medical science students, and those with applications for learning from healthcare providers. The respondent's inclusion or exclusion was decided in a brief section at the start of the questionnaire.

The questionnaire was tested for content validity by a panel of public health, women's health, and family medicine experts, and they reported the questions were suitable for the aim of the study. The questionnaire was tested for validity and reliability through a pilot study. The respondents confirmed the simplicity and clarity of the questions, and Cronbach's alpha was 0.78. Participants were notified that their responses had already been submitted to prevent duplicate submissions. Only responses with all the necessary data without any missed answers were used to analyze the results.

Sample size

The sample size was estimated using epi-info online software. The sample size was calculated to be 395. The participants were recruited using a volunteer sampling technique, and the number was increased to 539 to make the generalization of the results on the target population more acceptable.

Data collection tool

A self-administered electronic questionnaire (see Appendix) was designed on Google Forms (Google, Inc., Mountain View, CA), and the link to the questionnaire was distributed to individual accounts, in addition to groups, through various social networking platforms, like Facebook, Twitter, Snapchat, WhatsApp, and Instagram.

The questionnaire form consisted of 18 questions divided into two sections. The first section asked about the socio-demographic characteristics of the studied population (age, sex, education, marital status, nationality, and type of hospital where the participant receives their medical care, private or governmental). The second section included questions about the source of knowledge about GDM, whether the participant heard about GDM or not, and if they knew someone with GDM), risk factors, complications, prevention, and treatment of GDM.

The total knowledge score was calculated by summing all correct answers and dividing them by the total number of questions. Then, the level of knowledge was classified based on the four quartiles into poor (<25%), fair (25%-50%), good (50%-75%), and excellent levels of knowledge (>75%).

Data management and statistical analysis

The data were coded and recorded using Microsoft Excel (version 2019, Microsoft Corp., Redmond, WA). The data were analyzed statistically using Statistical Package for Social Sciences (SPSS) software (version 25, IMB Inc., Armonk, NY). Cronbach's alpha was used to test the validity and reliability of the questionnaire, and a value of more than 0.7 was considered acceptable. Descriptive statistics were calculated and presented as frequencies and percentages, mean ± SD. The multivariate linear regression analysis was employed to analyze the potential predictors of the high level of knowledge about GDM among the study population. A p-value of 0.05 or less was considered statistically significant based on the level of confidence of 95%.

Ethical consideration

Before starting to respond to the questionnaire, each participant had to provide consent indicating their permission to participate in the study. An introductory section of the questionnaire notified the participants about the purpose of the study, voluntary participation, and the confidentiality of the data collected, which would only be utilized for the current study. Additionally, all responses were collected anonymously, and the data was coded. No rewards were provided for taking part in the study.

Ethical approval was obtained from the ethical committee of Dr. Soliman Fakeeh Hospital (129\IRB\2020). All information in the study has been kept confidential and accessed only for scientific research purposes.

## Results

A total of 539 participants completed the questionnaire, with about half of them in the age category of 18-25 years) - 81.6% of them were females, more than half had a bachelor's degree, about half of them were single, and almost two-thirds of them were Saudi and attending private hospitals for health care (Table 1).

**Table 1:** General characteristics of the studied participants (n=539)

General characteristics		Frequency	Percent
Age	18-25	263	48.8
	26-35	103	19.1
	36-45	108	20.0
	46-55	55	10.2
	>56	10	1.9
Sex	Male	99	18.4
	Female	440	81.6
Education	Middle school	27	5.0
	High school	183	34.0
	Bachelor's degree	307	57.0
	Master's degree	16	3.0
	PhD	6	1.1
Marital status	Married	243	45.1
	Single	275	51.0
	Divorced	14	2.6
	Widowed	7	1.3
Nationality	Saudi	369	68.5
	Arabian gulf	138	25.6
	Non-Arabian countries	32	5.9
Hospital	Private	357	66.2
	Governmental	182	33.8

**Figure 1:**
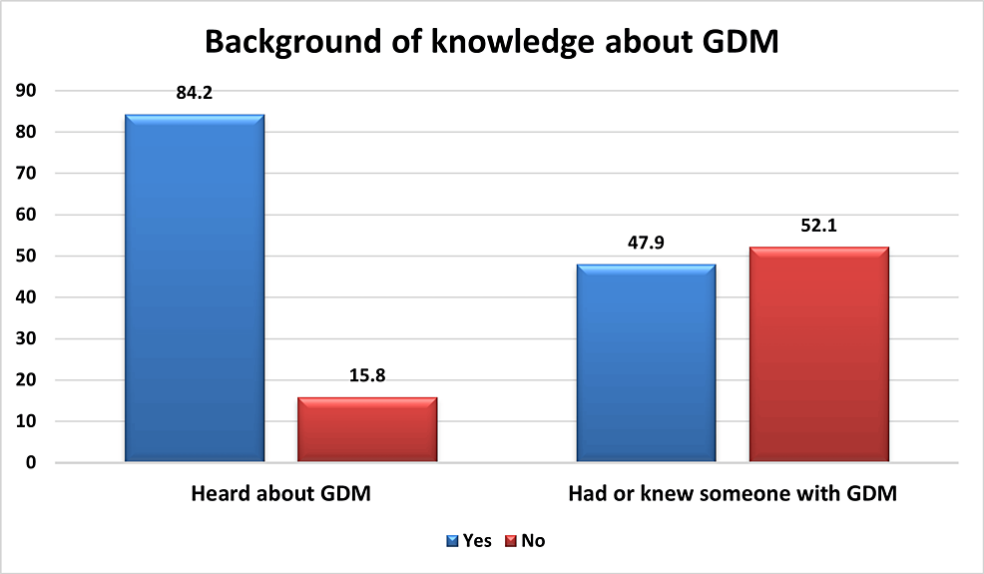
Background of knowledge about GDM among the studied participants (n=539) GDM - gestational diabetes mellitus

A total of 84.2% of the studied participants had heard about GDM, and 47.9% of them had or knew someone with GDM (Figure 1).

Most of the participants correctly answered about risk factors, consequences, and treatment of GDM (Table 2).

**Table 2:** Knowledge about GDM among the studied participants (n=539) GDM - gestational diabetes mellitus; T2DM - type 2 diabetes mellitus

Questions about knowledge	Yes, n (%)	No, n (%)	Don't know, n (%)
Do you think a healthy lifestyle can prevent GDM?	439 (81.4)	42 (7.8)	58 (10.8)
Do you think a family history of T2DM, obesity, or high maternal age can increase the risk of GDM?	393 (70.3)	73 (13.5)	87 (16.1)
Do you think females with GDM must have a blood sugar test performed after delivery?	479 (88.9)	19 (3.5)	41 (7.6)
Do you think that the risk of Cesarean delivery is higher among females with GDM?	255 (47.3)	87 (16.1)	197 (36.5)
Do you think the risk of stillbirth is higher among females with GDM?	203 (37.7)	77 (14.3)	259 (48.1)
Do you think females with GDM are at higher risk of T2DM in the future?	360 (66.8)	37 (6.9)	142 (26.3)
Do you think that the risk of hyperglycemia, weight gain, and type 2 DM is higher among infants of mothers with GDM?	509 (94.2)	15 (2.8)	16 (3)
Do you think diet, exercise, and insulin can be used for the treatment of GDM?	453 (82.9)	43 (8)	49 (9.1)
Do you recommend having an educational program about GDM for women?	495 (91.8)	9 (1.7)	35 (6.5)

**Figure 2:**
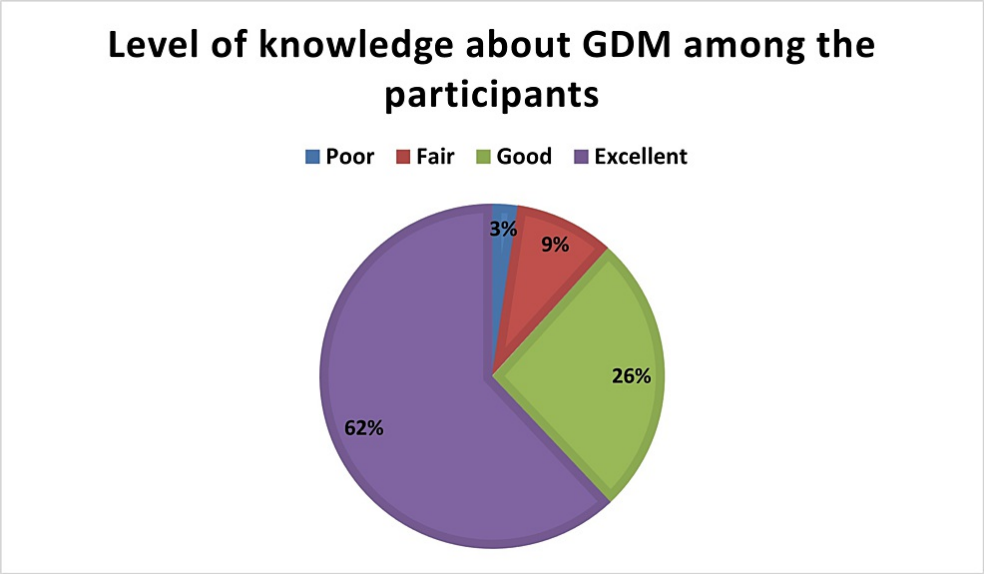
Level of knowledge about GDM among the studied participants (n=539) GDM - gestational diabetes mellitus

The total knowledge score was calculated and revealed that most of the studied participants had an excellent level of knowledge about GDM (62%), while the knowledge level was good among 26%, fair among 9%, and poor among 3% of participants (Figure 2).

In the multivariable linear regression model for the potential predictors of a high level of knowledge of GDM among the studied participants, receiving health care from governmental hospitals, ever heard about GDM, and having or knowing someone with GDM were positively associated with higher level of knowledge (β=0.097, CI: 0.006- 0.085; β=0.177, CI: -0.167-0.048; β=0.167, CI: 0.112-0.035, respectively; see Table 3).

**Table 3:** Multivariate regression analysis for the level of knowledge about GDM among the studied participants (n=539) GDM - gestational diabetes mellitus * p-value is statistically significant

Independent variables	Unstandardized coefficients		Standardized coefficients	t	p-value	95% confidence interval for B	
	B	Standard error	Beta			Lower bound	Upper bound
Age	-0.009	0.009	-0.046	-0.990	0.323	-0.027	0.009
Sex	0.052	0.027	0.092	1.909	0.057	-0.002	0.106
Education	0.023	0.014	0.070	1.682	0.093	-0.004	0.049
Marital status	-0.030	0.017	-0.083	-1.779	0.076	-0.063	0.003
Nationality	0.006	0.016	0.016	0.370	0.711	-0.026	0.038
Hospital	0.045	0.020	0.097	2.270	0.024*	0.006	0.085
Heard about GDM	0.107	0.030	0.177	3.547	0.0001*	0.167	0.048
Had GDM or know someone with GDM	0.074	0.020	0.167	3.751	0.0001*	0.112	0.035

## Discussion

The current study was conducted to assess the level of knowledge about GDM among the population in KSA. Five hundred and thirty-nine participants were recruited. Analysis of their responses revealed that the overall knowledge about GDM was excellent among 62% of participants, 26% showed a good level of knowledge; however, 9% had fair knowledge, and 3% had poor knowledge.

Despite having 62% of participants with excellent levels of GDM awareness, still a high percentage of respondents gave wrong answers to very important questions. For example, regarding the prevention of GDM, 18.6% of the participants didn't know that a healthy lifestyle can prevent GDM. The impact of lifestyle modifications, including physical exercise and dietary management, on the prevention and even treatment of GDM has been proven by multiple studies [9]. Healthy meal choices and appropriate food habits before and during pregnancy are protective against GDM [10]. Additionally, the involvement of females with GDM in aerobic training in combination with dietary modifications is considered the first-line treatment of GDM [11]. The lack of awareness about the importance of a healthy diet and physical exercise during pregnancy makes pregnant females at high risk of not only GDM but also numerous other diseases, including gestational hypertension, eclampsia, pre-eclampsia, infections, increased risk of cesarean section, hernia, and postpartum hemorrhage [12]. This highlights the importance of raising the awareness of pregnant females and the whole community about the importance of following a healthy lifestyle during pregnancy to reduce the risk of these hazardous risks. 

Assessment of the knowledge about the risk factors showed that 29.6% couldn't recognize that a family history of T2DM, obesity, or high maternal age can increase the risk of GDM. Earlier studies showed similar inappropriate levels of knowledge about GDM risk factors in Ethiopia [13] and Nigeria [14]. Bashir et al. (2022) [15] showed that the major risk factors of GDM include obesity, a history of previous GDM or previous delivery of a newborn weighing more than 4000 g, advanced maternal age, excessive weight gain during early pregnancy, a family history of overt diabetes, and previous diagnosis with polycystic ovary syndrome.

Appropriate knowledge about the risk factors of GDM is crucial for the management of this disease to minimize the feto-maternal risks because the guidelines emphasize the importance of having a blood glucose test at the beginning of pregnancy for females with risk factors; however, females with no risk factors are requested to have this test at 24^th^ week of gestation [16].

Evaluation of the knowledge about the maternal complications associated with GDM showed that 35.2% of the respondents didn't know that GDM can increase the risk of T2DM in the future, and 11.1% didn't know that females with GDM should have a test for blood sugar level after delivery. Regarding the awareness of the fetal complications of GDM, 5.8% of participants couldn't recognize any of the fetal complications like weight gain, hyperglycemia, or T2DM. Stillbirth is a major complication that can complicate GDM [17], even though most respondents (62.4%) didn't know about the correlation between GDM and this catastrophic complication.

The complications of GDM include, in addition to the maternal complications, numerous fetal complications like macrosomia, delivery trauma, shoulder dystocia, hyperbilirubinemia, neonatal hypoglycemia, and stillbirth [18]. Evaluation of the knowledge about the available treatment modalities of GDM showed that 17.6% of the participants didn't know about any of them. Despite these problems identified in the level of knowledge of the studied group, 8.2% of them didn't recognize the importance of educational programs about GDM.

Previous research studies showed large variations in the level of awareness of GDM among different communities. Some studies revealed appropriate levels of awareness; for example, the participants with a good level of awareness represented 76.1% in a study conducted in Tabuk City, KSA [19], 69.6% in Sohag, Egypt [20], and 73.5% in Sharjah [21]. 

On the other hand, other studies revealed inappropriate levels of awareness about GDM. For example, the participants with a good level of awareness represented 6.6% in the Qassim region [22], 2.2% in the Al-Baha area [23], and 7.80% in Al Madinah Al Munawara [1]. Similar results were identified outside KSA, where the population with appropriate awareness represents 31 % of participants in Uganda [24], 21.8% in Karnataka, India [25], and only 0.5% in Lebanon [26].

This diversity of the level of knowledge among studies can be attributed to multiple factors, including the variability of the socio-economic levels, the age and level of education of participants, the availability of awareness campaigns and counseling programs, the sample size, and the method of recruitment of participants.

The multivariate regression analysis test was employed to analyze the relationship between various demographic variables and the level of knowledge about GDM among the study population. The main factors that were positively correlated with a higher level of knowledge included previous diagnosis with GDM, knowing someone, or hearing about GDM, in addition to receiving treatment in governmental hospitals. On the other hand, no significant impact of age, sex, education level, marital status, and nationality on the level of knowledge was identified.

Refinement of awareness about GDM is crucial, as evidenced by a contemporary study conducted in China, which revealed that the level of knowledge about GDM among the studied population was positively correlated with the attitude and practice scores. Females with high knowledge scores had a better attitude towards the importance of monitoring diet and blood glucose levels, the nutritional counseling role during pregnancy, and the drug therapy for diagnosed GDM. They also showed better practices like monitoring diet, weight, blood glucose level, and compliance with the recommendations of doctors and dieticians [27]. 

These findings shed light on the importance of having awareness campaigns about risk factors, complications, and treatment of GDM. Awareness campaigns are recommended for the entire population, with more emphasis on females of childbearing age. Additionally, private hospitals should consider conducting counseling sessions to improve awareness about GDM.

A recent study conducted in Italy by Quaresima et al. (2021) [4] revealed that counseling sessions can cause a significant increase in the level of awareness about GDM among pregnant females. In Australia, standard education with or without web-based educational programs was found to result in excellent knowledge scores among females with GDM [28]. 

Limitations of the study

Due to the use of an online survey and a self-administered questionnaire for data collection, there were certain limitations to the current study. Only those with reading proficiency in Arabic or English were able to reply to this survey. The questionnaire was not accessible to those without access to social media and the internet. Additionally, the study participants' access to social media platforms and the internet exposes them to various educational resources that can increase their knowledge of health-related topics, including GDM, more than the rest of the population. Because the respondents self-administered the questionnaire, it is possible that they misinterpreted some of the questions. Due to these factors, the study population selected by convenience sample does not accurately reflect the target population, so the generalization of the findings may not be applicable. Moreover, because the study design is cross-sectional, there is little precision in determining the relationship between the different studied variables. Nevertheless, it can offer preliminary data that can aid in the development of a comprehensive perspective regarding the community's awareness of such a crucial health-related issue and the subsequent development of appropriate projects aimed at increasing population awareness and enhancing overall public health.

## Conclusions

The findings revealed that among participants, 334 (62%) showed excellent knowledge about GDM, although the other 205 (38%) had non-optimal levels of knowledge. Participants with less than 75% score represented 38% of the studied group. Excellent knowledge was more common among participants who heard about or knew someone with GDM and those who received treatment in governmental hospitals. Awareness campaigns are recommended to improve the level of knowledge about this disease, its risk factors, treatment, and complications.

## Appendices

Questionnaire to assess the awareness of gestational diabetes mellitus.

1. Name (optional)

2. Gender

a. Male

b. Female

3. Age

a. 15-25

b. 26-35

c. 36-45

d. 46-55

e. ≥56

4. Educational level

a. Elementary

b. Middle school

c. High school

d. Bachelor's degree

e. Master's degree

f. PhD

5. Marital status

a. Married

b. Single

c. Divorced

d. Widowed

6. Nationality

a. Saudi

b. Arabian Gulf

c. Non-Arabian countries

7. What hospital are you visiting?

a. Private hospital

b. Governmental hospitals

8. Have you ever heard about gestational diabetes mellitus?

a. Yes

b. No

9. Did you have or know someone with gestational diabetes mellitus?

a. Yes

b. No

10. Do you think females with gestational diabetes mellitus must have a blood sugar test performed after delivery?

a. Yes

b. No

c. I don't know

11. Do you think the risk of cesarean delivery is higher among females with gestational diabetes mellitus?

a. Yes

b. No

c. I don’t know

12. Do you think the risk of stillbirth is higher among newborns of mothers with gestational diabetes mellitus?

a. Yes

b. No

c. I don't know

13. Do you think females with gestational diabetes mellitus are at higher risk of diabetes mellitus type II in the future?

a. Yes

b. No

c. I don't know

14. Do you think a healthy lifestyle can decrease the risk of gestational diabetes mellitus?

a. Yes

b. No

c. I don't know

15. Do you think that a family history of diabetes mellitus, obesity, high maternal age, or previous gestational diabetes mellitus can increase the risk of gestational diabetes mellitus in pregnant females?

a. Yes

b. No

c. I don't know

16. Do you think the risks of glucose intolerance, weight gain, and diabetes mellitus are higher among infants born to mothers with gestational diabetes mellitus?

a. Yes

b. No

c. I don't know

17. Do you think that diet, exercise, and insulin therapy, can be used for the treatment of gestational diabetes mellitus?

a. Yes

b. No

c. I don't know

18. Do you recommend having an educational program about gestational diabetes mellitus?

a. Yes

b. No

c. I don't know
